# Comment on “Xerostomia and Salivary Gland Hypofunction in Patients with Oral Lichen Planus Before and After Treatment with Topical Corticosteroids”

**DOI:** 10.2174/1874210601812010310

**Published:** 2018-04-30

**Authors:** Mahdieh-Sadat Moosavi, Hoda Barati

**Affiliations:** Dental Research Center, Department of Oral Medicine / School of Dentistry, Tehran University of Medical Sciences, Tehran, Iran

## DEAR EDITOR

The recently published research article “Xerostomia and Salivary Gland Hypofunction in Patients with Oral Lichen Planus Before and After Treatment with Topical Corticosteroids” by Hala Al-Janaby, *et al*. [1] was studied with great interest. The article concluded that treatment with topical corticosteroids in patients with oral lichen planus could result in improving the symptoms of xerostomia. The treatment, however, is not statistically associated with differences in stimulated and unstimulated salivary flow rate, unstimulated salivary pH or buffering capacity. Since the two conditions (xerostomia and salivary gland hypofunction: which these terms are not equal, Xerostomia: the subjective complaint of dry mouth. Salivary gland dysfunction: objective evidence of alterations (qualitative and/or quantitative) in saliva output. Dysfunction may include a decrease (hypofunction) or an increase (hyperfunction) of the saliva output (Mahvash Navazesh 2017) are commonly observed in patients with oral lichen planus [1], the results of the above study could significantly impact the management and treatment of those patients.With respect to the results presented in the article, the followings are of concern:

1. Most cases of OLP were diagnosed on clinical presentation alone (WHO clinical diagnostic criteria) with the remaining cases fulfilling both the WHO clinical and histopathologic criteria: According to recent studies [2, 3] one of the most common criteria used for the diagnosis of OLP is modified WHO which is based on clinical and histopathological criteria. If other clinical and histopathological criteria have been used for the diagnosis of OLP from other lichenoid lesions, it should be mentioned. This approach has not been adopted for most of the participants in the study. It should also be mentioned that some patients could develop symptoms of lichenoid reactions (LR) instead, and therefore, the results of the study could not be applicable to OLP, especially when considering the completely different etiopathogenesis of lichenoid reactions [1].
2. Out of 19 participants in this study who had OLP, 12 participants were taking medications that influence the salivary flow rate. Since the research objective was to study salivary flow rate and xerostomia, it is expected that all influencing factors were either taken out of the study or investigated separately in another group. If the reduced salivary flow rate and development of xerostomia are associated with the medications that participants were taking, which is very likely, the results of the study could not be directly associated with the treatment of OLP and its effects on the salivary flow rate and xerostomia. It is even possible that by excluding participants who had taken medications to reduce salivary flow rate, the flow rate after treatment with TC in OLP patients varies much, as the factor that causes mouth dryness was present before and after treatment [1].
3. Samples of saliva were collected between 9:00 am to 12:00 pm or 1:00 am to 4:00 pm as described in Materials and Methods section of the article. Since circadian rhythm is completely dependent on the time when samples were collected and it also affects the salivary secretion [4], various salivary flow rates among samples is expected. The research does not take this into account.
4. There were 7 participants with reticular type and 12 participants with ulcerative/erosive pattern in the study. Now the question is whether the type of corticosteroid medication (short acting, intermediate acting, and long acting) and the amount and the number of times it was taken were the same? Because, it is obvious that the type of treatment directly affects the improvement in the patient conditions and the reduction of the symptoms of SFR and xerostomia [5, 6].

## References

[1] Al-Janaby H., El-Sakka H., Masood M., Ashani W Mendis W., M Slack-Smith L., Parsons R., M Frydrych A. (2017). Xerostomia and salivary gland hypofunction in patients with oral lichen planus before and after treatment with topical corticosteroids.. Open Dent. J..

[2] Alrashdan M.S., Cirillo N., McCullough M. (2016). Oral lichen planus: A literature review and update.. Arch. Dermatol. Res..

[3] van der Meij E.H., van der Waal I. (2003). Lack of clinicopathologic correlation in the diagnosis of oral lichen planus based on the presently available diagnostic criteria and suggestions for modifications.. J. Oral Pathol. Med..

[4] Dawes C. (1972). Circadian rhythms in human salivary flow rate and composition.. J. Physiol..

[5] Siponen M., Huuskonen L., Kallio-Pulkkinen S., Nieminen P., Salo T. (2017). Topical tacrolimus, triamcinolone acetonide, and placebo in oral lichen planus: A pilot randomized controlled trial.. Oral Dis..

[6] Villa A., Wolff A., Aframian D., Vissink A., Ekström J., Proctor G., McGowan R., Narayana N., Aliko A., Sia Y.W., Joshi R.K., Jensen S.B., Kerr A.R., Dawes C., Pedersen A.M. (2015). World Workshop on Oral Medicine VI: A systematic review of medication-induced salivary gland dysfunction: prevalence, diagnosis, and treatment.. Clin. Oral Investig..

